# Risk factors for coronary heart disease in patients below 45 years of age

**DOI:** 10.12669/pjms.291.2828

**Published:** 2013

**Authors:** Mansoor Nadeem, Syed Shahzad Ahmed, Sarah Mansoor, Sidra Farooq

**Affiliations:** 1 Mansoor Nadeem, MBBS, MCPS, FCPS, Associate Professor, Department of Medicine, POF Hospital, Wah Medical College, Wah Cantonment, Punjab, Pakistan.; 2 Syed Shahzad Ahmed, MBBS, House Officer, Department of Medicine, POF Hospital, Wah Medical College, Wah Cantonment, Punjab, Pakistan.; 3 Sarah Mansoor, MBBS, House Officer, Department of Radiology, POF Hospital, Wah Medical College, Wah Cantonment, Punjab, Pakistan.; 4 Sidra Farooq, MBBS, PGT FCPS Part II, Department of Medicine, POF Hospital, Wah Medical College, Wah Cantonment, Punjab, Pakistan.

**Keywords:** Risk Factors, Coronary Artery Disease

## Abstract

***Objective: ***To examine the traditional risk factors and biochemical profile of patients with established CAD (coronary artery disease), and compare the trends of these in specified age groups of different populations as depicted in various studies.

***Methodology:*** All consecutive patients below 45 years of age, having classical history of Ischemic heart disease and also having definite ECG changes consistent with coronary artery disease were enrolled. These patients were admitted to CCU/Intermediate Coronary Care Unit of Pakistan Ordinance Factories (POF) Hospital Wah Cantonment from April 2007 to December 2011. Patients who had doubtful history as regards CHD and those having ECG changes not classically consistent with CAD were excluded. Information collected through Performa included history including family history and details of risk factors. Clinical examination was carried out and relevant investigations including the serial ECG changes were recorded. Blood samples were collected after an overnight fast of 14 hours and tests were done for total cholesterol and HDL cholesterol by using Pioneer-USA, linear chemical kits by cholesterol oxidase and enzymatic calometric method.

***Results: *** A total of 109 cases were included. Cigarette smoking (46%) Family history (43%), Hypertension (37%), Dyslipidemia (33%), Diabetes mellitus (18%) and above normal BMI (63.3%) are the most common risk factors in our patients. Increased abdominal girth has appeared to be an important risk factor and at occasions is documented to be independent of obesity. Casual dietary habits and sedentary life style are the other less important risk factors. The majority of risk factors were equally prevalent in males as well as females except smoking which was less prevalent in females.

***Conclusions: ***Our study shows that Family history, Smoking, Hypertension, increased BMI, increased Abdominal girth, Dyslipidemia and Diabetes Mellitus are the main risk factors. Considering the increasing incidence of the coronary heart disease in our society it is essential to assess and evaluate these risk factors at national level.

## Introduction

 Coronary artery disease (CAD) remains the commonest cause of mortality world wide.^1^ South Asians are prone to develop advanced atherosclerosis and highest mortality rate due to its complications compared to other ethnic groups studied.^2^ It has been documented that younger age group people are developing the diseases frequently than before in last couple of years.^3^ Various studies show that in comparison with older patients these patients have single vessel disease^4^, has more profound hypercholesterolemia^4^, significant positive family history^4^ and history of smoking.^5^ There are a number of such studies from developed countries but only a few from Pakistan.

 Coronary heart disease mortality is generally accepted as an indicator of socio-economic conditions. In a changing social environment, early detection and treatment of youths at risk of premature IHD offers the greatest promise and an opportunity for age-specific interventions.^6^ Moreover, the potential gains from controlling major established risk factors could be substantial in South Asians and greater than that in Europeans.^7^

 The purpose of this study was to evaluate different risk factors prevailing in patients below 45 years of age suffering from coronary heart disease.

## Methodology

 All consecutive patients age below 45 years, having classical history of Ischemic heart disease and also having definite ECG changes consistent with coronary artery disease (recent / old) were enrolled. These patients were admitted to CCU/Intermediate Coronary Care Unit of Pakistan ordinance factories (POF) Hospital Wah Cantonment from April 2007 to December 2011. POF Hospital Wah Cantonment is 620 bedded tertiary care centre for the employees and their dependents, civilians living at POF Wah cantonment and tertiary care referral hospital for POF Sanjwal cantonment and POF Havelian cantonment.

 In total one hundred and nine (109) patients were included in the study. Patients who had doubtful history as regards coronary heart disease and those having ECG changes not classically consistent with CAD were excluded. The patients were divided into 4 groups on the bases of age; group1, <30 years; group2, between 31-35 years; group3, between 36-40 years and group 4 between 41- 45 years.

 A detailed comprehensive Performa was filled by our trained doctors regarding history including family history and details of risk factors. Clinical examination was carried out and relevant investigations including the serial ECG changes were recorded. Blood samples were collected after an overnight fast of 14 hours and tests were done for total cholesterol and HDL cholesterol by using Pioneer-USA, linear chemical kits by cholesterol oxidase and enzymatic calometric method.

 The patients were considered suffering from hypertension once the patient was on antihypertensive medications at the time of admission or the past medical history / record documented raised BP at multiple occasions or the BP was recorded higher (>140/90 mmHg) at separate occasions while remained admitted. 

 Diabetes Mellitus was considered to be present when either patient was taking any anti-diabetic agents or blood glucose was found to be > 126 mg/dl fasting or > 200 mg/dl at random sample at more than 02 occasions.

 Cigarette smoking was labeled if the patient had smoked within last 03 years. Family history of ischemic heart disease was considered positive when any close relative <55 years of age in males or <65 years in female had history of angina pectoris or myocardial infarction in past.

 Dyslipidemia was considered when total cholesterol was found more than 5.2 - 6.5mmol/L (200 mg/dl) and high density lipoproteins (HDL) <1.0 mmol/L (≤ 40 mg/dl). The data was processed on SPSS Statistics v16.

## Results

 The study comprised of 109 patients with mean age 41 years. There were 67 males (61%) and 42 females (39%) (Table-I). Males mean age was 42 years and females mean age was 41 years. Further divisions of patients were made according to age in 4 groups. Group 1, age < 30 years had a total of 04 (02 males & 02 female). Group 2, ages from 31- 35 years had a total of 11 (08 males and 03 females), group 3, ages from 36-40 years had a total of 24 (12 males and 12 females). Group 4, ages from 41- 45 years had a total of 70 (males 45 and females 25). Majority of the patients were in group 4 (64.2%). (Table-I)

**Table-I:** Distribution according to gender & age

		*Total*		*Gender*			
				*Male*		*Female*	
		*Count*	*%*	*Count*	*Table N %*	*Count*	*Table N %*
Age Category (years)	<=30	4	3.7%	2	1.8%	2	1.8%
	31-35	11	10.1%	8	7.3%	3	2.8%
	36-40	24	22.0%	12	11.0%	12	11.0%
	41-45	70	64.2%	45	41.3%	25	22.9%
Total				67	61.5%	42	38.5%

**Table-II:** Distribution of risk factors according to gender

*S. No.*			*Gender*				*Total*	
			*Male*		*Female*			
			*Count*	*Table N%*	*Count*	*Table N%*	*Count*	*%*
a.	Smoking	Yes	47	43.1%	3	2.8%	50	45.9%
		No	20	18.3%	39	35.8%	59	54.1%
b.	Family History	Positive	28	25.7%	19	17.4%	47	43.1%
		Negative	39	35.8%	23	21.1%	62	56.9%
c.	Hypertension	Yes	22	20.2%	18	16.5%	40	36.7%
		No	45	41.3%	24	22.0%	69	63.3%
d.	Dyslipidemias	Yes	21	19.3%	12	11.0%	33	30.3%
		No	46	42.2%	30	27.5%	76	69.7%
e.	Diabetes Mellitus	Yes	15	13.8%	5	4.6%	20	18.3%
		No	52	47.7%	37	33.9%	89	81.7%
f.	BMI (Kg/m2)	<18.50	1	0.9%	0	0.0%	1	0.9%
		Normal (18.5-24.9)	30	27.8%	9	8.3%	39	35.8%
		Overweight (25-29.9)	25	23.1%	24	22.2%	49	45.0%
		Class I Obesity (30-34.9)	8	7.4%	9	8.3%	17	15.6%
		Class II Obesity (35-39.9)	1	0.9%	0	0.0%	1	0.9%
		Class III Obesity >40	1	0.9%	0	0.0%	1	0.9%
g.	Abdominal Girth (inches)	31-35	18	18.6%	2	2.1%	20	18.3%
		36-40	30	30.9%	34	35.1%	64	58.7%
		41-45	8	8.2%	3	3.1%	11	10.1%
		46-50	2	2.1%	0	0.0%	2	1.8%
h.	Diet	Good	29	26.6%	13	11.9%	42	38.5%
		Bad	38	34.9%	29	26.6%	67	61.5%
i.	Lifestyle	Active	29	26.6%	5	4.6%	34	31.2%
		Sedentary	38	34.9%	37	33.9%	75	68.8%
j.	Work/Job	Executive	10	9.2%	1	0.9%	11	10.1%
		Office work	24	22.0%	4	3.7%	28	25.7%
		Manual work	32	29.4%	0	0.0%	32	29.4%
		Housewife	0	0.0%	38	34.0%	38	34.9%

 Smoking was documented in 50 patients (45.9%). Forty seven (43.1%) were males and only 3 (2.8%) were females. Eighty percent of the smokers were in age group 4. Strong family history for CAD was noted in 47 patients (43.1%), 28 (25.7%) were males and 19 (17.4%) was females. (Table II & III)

 Hypertension was noted in 40 patients (36.7%). Twenty two (20.2%) were males and 18 (16.5%) were females. Seventy five percent of the hypertensive patients were in age group 4. Dyslipidemia was noted in 33 (30.3%) patients and of these 21 (19.3%) were males and 12 (11%) females. Average serum cholesterol was 5.85 mmol/L and HDL was 1.005 mmol/L. Fifty eight percent of the patients with dyslipidemias were in age group 3 and 15% of patients were in group 2. Diabetes Mellitus was noted in 20 patients (18.3%). Males were 15 (13.8%) and females 5 (4.6%). Seventy percent of the diabetics were in age group 4. (Table II & III)

**Table-III:** Distribution of risk factors according to age group

*S. No.*			*Age Category (years)*							
			*<=30*		*31-35*		*36-40*		*41-45*	
			*Count*	*Table N %*	*Count*	*Table N %*	*Count*	*Table N %*	*Count*	*Table N %*
a.	Smoking	Yes	1	0.9%	2	1.8%	7	6.4%	40	36.7%
		No	3	2.8%	9	8.3%	17	15.6%	30	27.5%
b.	Family History	Positive	1	0.9%	5	4.6%	11	10.1%	30	27.5%
		Negative	3	2.8%	6	5.5%	13	11.9%	40	36.7%
c.	Hypertension	Yes	2	1.8%	1	0.9%	7	6.4%	30	27.5%
		No	2	1.8%	10	9.2%	17	15.6%	40	36.7%
d.	Dyslipidemias	Yes	1	0.9%	5	4.6%	3	2.8%	24	22.0%
		No	3	2.8%	6	5.5%	21	19.3%	46	42.2%
e.	Diabetes Mellitus	Yes	0	0.0%	2	1.8%	4	3.7%	14	12.8%
		No	4	3.7%	9	8.3%	20	18.3%	56	51.4%
f.	BMI (Kg/m2)	<18.50	0	0.0%	0	0.0%	0	0.0%	1	0.9%
		Normal (18.5-24.9)	1	0.9%	5	4.6%	11	10.2%	22	20.4%
		Overweight (25-29.9)	3	2.8%	6	5.6%	7	6.5%	33	30.6%
		Class I Obesity (30-34.9)	0	0.0%	0	0.0%	6	5.6%	11	10.2%
		Class II Obesity (35-39.9)	0	0.0%	0	0.0%	0	0.0%	1	0.9%
		Class III Obesity (>40)	0	0.0%	0	0.0%	0	0.0%	1	0.9%
g.	Abdominal Girth (inches)	31-35	1	1.0%	2	2.1%	5	5.2%	12	12.4%
		36-40	3	3.1%	4	4.1%	12	12.4%	45	46.4%
		41-45	0	0.0%	0	0.0%	4	4.1%	7	7.2%
		46-50	0	0.0%	0	0.0%	0	0.0%	2	2.1%
h.	Diet	Good	2	1.8%	3	2.8%	9	8.3%	28	25.7%
		Bad	2	1.8%	8	7.3%	15	13.8%	42	38.5%
i.	Lifestyle	Active	2	1.8%	4	3.7%	7	6.4%	21	19.3%
		Sedentary	2	1.8%	7	6.4%	17	15.6%	49	45.0%
j.	Work/Job	Executive	1	0.9%	2	1.8%	0	0.0%	8	7.3%
		Office work	0	0.0%	4	3.7%	8	7.3%	16	14.7%
		Manual work	1	0.9%	2	1.8%	5	4.6%	24	22.0%
		Housewife	2	1.8%	3	2.8%	11	10.1%	22	20.2%

 Forty five percent were overweight with BMI 25-29.9 Kg/m2. Majority of these patients were in age groups 3 and 4. A reasonable number of patients (15.6%) had class I obesity (BMI 30- 34.9 Kg/m2). Only 35.8% patients had normal weight and had normal BMI (18-24.9 Kg/m2). (Table-IV)

 Abdominal girth was noted to be an important risk factor and at times operate independent of obesity (Increased BMI). Fifteen cases with 36-40 inches abdominal girth were noted with normal BMI (18-25 Kg/m2), 37 patients had girth of 36-40 inches while having BMI 26-30 Kg/m2 and 08 patients had abdominal girth of 36 - 41 inches while having BMI of 26-30 Kg/m2. Only 04 patients had 41-45 inches of abdominal girth. (Table-IV)

**Table-IV:** Body Mass Index (BMI) vs Abdominal Girth

				*Count*
BMI (Kg/m2)	<= 18.50	Abdominal Girth (inches)	31-35	1
			36-40	0
			41-45	0
			46-50	0
	Normal (18.5-24.9)	Abdominal Girth (inches)	31-35	23
			36-40	15
			41-45	1
			46-50	0
	Overweight (25-29.9)	Abdominal Girth (inches)	31-35	8
			36-40	37
			41-45	4
			46-50	0
	Class I obesity (30-34.9)	Abdominal Girth (inches)	31-35	0
			36-40	11
			41-45	5
			46-50	1
	Class II obesity (35-39.9)	Abdominal Girth (inches)	31-35	0
			36-40	0
			41-45	1
			46-50	0
	Class III Obesity >40	Abdominal Girth (inches)	31-35	0
			36-40	0
			41-45	0
			46-50	1

 Casual dietary habits were noted in 67 (61.5%), of these 38 (34.9%) were males and 29 (26.6%) were females. Eighteen percent patients with casual dietary habits had BMI in normal limits while 42% of patients had increased BMI above the normal (<24.9Kg/m2).

 Active lifestyle was noted in 34 (31.2%) patients, 29 (26.6%) were males and 5 (4.6%) were females. Majorities (68.8%) of the patients had sedentary life style and were distributed equally in both sexes.

 Thirty four percent of males and only 5% females were employed in executive or sedentary office jobs. Thirty eight (95%) females were housewives. Thirty two patients were manual workers and belonged to lower socio-economic class.

## Discussion

 The prevalence of coronary heart disease has been tremendously increasing in last decade. In this study there were 61% males compared to 39% females. In studies done earlier in Pakistan the female population was lower which was probably due to sample selection bias as the patients were recruited from angiography units and not from the coronary care units.^8^^,^^9^

 Smoking is important and modifiable risk factor known for CAD.^10^ Our data shows that the smoking was the dominant risk factor (46%). Only 3% females smoked. Several other studies have shown smoking as the most important risk factor among the younger patients with CAD.^11^ Pais et al has shown it to be the most dominant risk factor in Indian population studied.^12^ Our study also demonstrates that cigarette smoking was the dominant risk factor predisposing to an earlier onset of CAD, in line with previous studies.^13^^,^^14^

 The Framingham study shows the mechanism of this correlation; that cigarette smoking is strongly associated with “atherogenic” lipoprotein cholesterol profiles in young adults.^15^ This signifies the fact that cessation of smoking may be the most cost effective approach in primary and secondary prevention.

 We have observed that hypertension was the second commonest risk factor (37%) and was equally distributed in both sexes. The exact mechanism through which systemic hypertension induces MI has not been studied in detail, but there is evidence that Hypertension causes LV Hypertrophy and progression of atherosclerosis resulting in CAD.^16^

 Diabetes mellitus was noted in 17%. The disease is more prevalent in males (14%) compared to females (4%) and in older patients as compared to younger patients. This fact has been documented in a number of previous studies.^13^^,^^14^

 Higher BMI was documented in 46% of patients. Sixteen were obese. There was no statistical difference comparing males with females. Asians have a higher body fat percentage for a given BMI than other ethnic groups. Prevalence of obesity and overweight is low, while DM and HTN occur at a lower level of BMI compared to western population. It has been suggested that Asians have a different fat distribution pattern and are more prone to central obesity at low BMI levels.^17^

 Increased cholesterol was noted in 33 (33%) patients. Hypercholesterolemia mainly increased LDL and decreased HDL are important risk factors. It is documented that South Asians have total cholesterol, LDL-C levels comparable to Afro-Caribbean’s and whites but they do have higher Triglycerides and low HDL-C levels.^12^ In another study done in India has shown that mean TC in Indians is usually below 200 mg/dl^12^ but ratio of TC/HDL-C is significantly lower.^14^ In our patients hypercholesterolemia was highest (73%) in group 4, which is comparable to the findings of Yildirim et al.^14^ In another study, no graded strength of association of hypercholestrolemia with coronary artery disease could be observed.^18^ It has also been observed that in South Asians, CAD occurs at a much lower level of total cholesterol than other populations.^19^

 The mechanism of such an observation could be explained by a study showing that the serum HDL cholesterol of South Asians is comparable to that of other populations, only the small HDL particle size is predominant in them, which does not confer protection against CAD.^20^

 Strong family history was noted in 47% of patients which was equally noted in both males and females and appears to be another important risk factor. This fact was also highlighted in another study done in India.^21^

 There have been several studies to explain the mechanism of this correlation. In a study to detect the cardiovascular risk in young adults, it was found that young subjects with a positive family history of CAD had greater sub-clinical atherosclerosis (IMT) compared to those with negative history. The association between the number of risk factors and IMT was stronger in subjects with a family history than those without.^22^ It has also been observed that subjects with a family history of CAD had higher prevalence of coronary artery calcium in the presence of metabolic risk factors than those without a family history.^23^

 Therefore overall evidence suggests that risk of atherosclerotic coronary vascular disease is increased among those with a family history of diabetes; suggesting that genetic factors associated with occurrence of familial diabetes may increase risk of CAD beyond the risk among people without family history of diabetes.^24^

 Life style of the patients was not an important risk factor as sedentary life style was noted in only 39% of patients and the majority were males. Similarly the nature of job also seems to be an un-important risk factor as the disease appears to be equally prevalent in persons employed at different jobs.

## Conclusions

 Our study shows that Family history, smoking, Hypertension, increased BMI, Increased abdominal girth, Dyslipidemia and Diabetes Mellitus are the main risk factors. There is no notable gender difference in frequency of various risk factors except in smoking which is almost exclusively noted in males. However there is very limited data available in the form of formal studies from our country. Considering the increasing incidence of the coronary heart disease in our society it is essential to assess and evaluate these risk factors prevalent at national level. It will enable us in formulating policies for promoting healthy lifestyles, frequent and early risk assessment and age specific preventive strategies.
